# Oleanolic Acid Induces Autophagy and Apoptosis via the AMPK-mTOR Signaling Pathway in Colon Cancer

**DOI:** 10.1155/2021/8281718

**Published:** 2021-07-14

**Authors:** Changxiao Hu, Yibo Cao, Ping Li, Xiaorong Tang, Minhui Yang, Shengliang Gu, Kai Xiong, Tian Li, Tianbao Xiao

**Affiliations:** ^1^College of Clinical Medicine, Guizhou University of Traditional Chinese Medicine, No. 71 Baoshan North Road, Guiyang 550001, China; ^2^Colorectal and Anal Surgery, The First Affiliated Hospital of Guizhou University of Traditional Chinese Medicine, No. 71 Baoshan North Road, Guiyang 550001, China; ^3^Fourth Military Medical University, No. 169 Changle West Rd, Xi'an 710032, China

## Abstract

**Aims:**

The purpose of this study was to explore the biological functions of the mTOR and AMPK signaling pathways in colon cancer (CC). The potential molecular mechanisms by which oleanolic acid (OA) induces autophagy and apoptosis were also investigated.

**Methods:**

The biological functions of mTOR were analyzed by GeneCards, the Search Tool for the Retrieval of Interacting Genes (STRING), and the Database for Annotation, Visualization and Integrated Discovery (DAVID). Least absolute shrinkage and selection operator (LASSO) regression analysis was used to obtain prognostic and survival data of CC patients from the Gene Expression Omnibus (GEO) database. The effects of OA on the CC cell lines HCT-116 and SW-480 were analyzed by CCK-8, colony formation assay, and high-content system (HCS) array scan. The apoptosis rate of SW-480 and HCT-116 cells was detected by flow cytometry. The mRNA and protein expression levels in HCT-116 and SW-480 cells and NCM-460 normal colonic epithelial cells were detected by RT-PCR and Western blotting.

**Results:**

mTOR was highly expressed in CC patients and acted as an oncogene. The AMPK signaling pathway mediated by mTOR predicted the poor prognosis of CC patients. OA effectively inhibited the proliferation and viability of CC cells. Furthermore, the apoptosis rate of CC cells was clearly increased following OA administration. Regarding the molecular mechanism of OA, the results indicated that mTOR and the antiapoptosis gene Bcl-2 were downregulated by OA. In addition, regulator genes of autophagy and apoptosis, including BAX, caspase-9, caspase-8, and caspase-3, were significantly upregulated by OA. Moreover, OA upregulated AMPK and its downstream proteins, including TSC2, BAX, Beclin 1, LC3B-II, and ULK1, to induce autophagy and apoptosis in CC cells.

**Conclusion:**

The findings from this study demonstrate that OA could effectively inhibit the proliferation and viability of CC cells. The anti-CC activity of OA is closely related to the activation of the AMPK-mTOR signaling pathway. Activation of AMPK and inhibition of mTOR are involved in the induction of autophagy and apoptosis by OA. OA induced autophagy and apoptosis mainly in an AMPK activation-dependent manner in CC cells.

## 1. Introduction

Colon cancer (CC) is one of the most common malignant tumors and the third most common cancer, after lung and prostate cancer, in the world [1]. According to global cancer data, there are approximately 1.1 million newly diagnosed CC patients and 550 thousand CC-related fatalities, and the rate of death from CC is predicted to increase by 60% in 2035 [2, 3]. Generally, surgery, radiotherapy, and chemotherapy are the main treatment options for patients with CC at present. However, only 70% of CCs are resectable, of which 75% are curable and 25% of resected patients will have recurrent tumors [4]. Even with the progress in early diagnosis and treatment, the 5-year relative survival rate of CC patients is still only 63% [1]. Moreover, increasing drug resistance and adverse effects related to radiotherapy and chemotherapy have severely degraded the quality of life of CC patients. Therefore, novel treatment strategies and drugs are urgently required to meet the clinical needs of CC patients.

Autophagy is a conserved, self-degradation system that is critical for maintaining cellular homeostasis during stress conditions. Dysregulated autophagy has implications in health and cancer. Specifically, in cancer, autophagy plays a dichotomous role by inhibiting tumor initiation but supporting tumor progression [5]. Like apoptosis, autophagy is a form of cell death [6]. Autophagy is mainly regulated by the AMPK/mTOR signaling pathway, PI3K/Akt/mTOR signaling pathway, and other related autophagy signaling pathways. As one of the classical signaling pathways in regulating autophagy, the AMPK/mTOR signaling pathway has provided many potential therapeutic targets for CC. mTOR, an atypical serine/threonine protein kinase protein, regulates cell growth and proliferation predominantly by promoting key anabolic processes, sensing nutrition levels and growth factors, as well as various environmental cues [7, 8]. Moreover, mTOR has been defined as an oncogene in CC and has emerged as an effective target for CC therapy in both *in vitro* and preclinical studies [9, 10]. In addition, the energy sensor adenosine monophosphate (AMP)-activated protein kinase (AMPK) plays a key role in maintaining energy homeostasis by sensing energy loss and activating cell autophagy [11]. Activation of AMPK can induce the expression levels of caspase-3, caspase-8, and caspase-9, leading to apoptosis in CC cells [12]. AMPK activation also stimulated autophagy by increasing the protein expression levels of Beclin 1 and LC3-II and decreasing the levels of p-mTOR and p-ULK1 in CC cells [13]. Furthermore, AMPK can negatively regulate the mammalian target of mTOR, thereby inducing autophagy and apoptosis of CC *in vivo* and *in vitro* [13, 14]. Constitutively, suppression of mTOR, as well as mTOR complex 1 (mTORC1), has been demonstrated in CC cells, enhancing the ability to modulate growth inhibition, proliferation, apoptosis, and autophagy of CC [14]. Therefore, regulation of the AMPK/mTOR signaling pathway could greatly contribute to the treatment of CC.

Oleanolic acid (3-*α*-3-hydroxyolean-12-en-28-oic acid, OA) is a natural pentacyclic triterpene carboxylic acid that exists widely in plant molecules. The anticancer activities of OA in various types of tumors, such as colorectal cancer, thyroid cancer, gastric cancer, breast cancer, and prostate cancer, have been explored [15, 16]. In colon cancer research, a previous study demonstrated that OA decreased the proliferation ability of HCT-15 cells by arresting the cell cycle in the G0/G1 phase and inhibiting DNA replication [17]. Another study revealed that OA induced strong G0/G1 cell cycle arrest and DNA fragmentation in HT-29 and Caco-2 cells. Additionally, OA led to mitochondrial apoptosis dependent on an increase in caspase-3 activity via a p53-independent mechanism [18]. In addition, the inhibition of HT-29 cell proliferation induced by OA occurred in a dose-dependent manner [19]. Another study indicated that OA and its analogs with apoptosis-inducing activity effectively inhibited the incidence of abnormal recess and multitree lesions in rats with colon cancer, indicating that OA and its analogs have a chemoprophylactic effect on colon cancer *in vivo* [20]. Simultaneously, numerous phase I clinical trials have reported that OA and its derivatives exhibit potent antitumor activities in advanced solid tumors with limited toxicities [21, 22]. Therefore, it is evident that OA is a promising therapeutic agent against CC.

In this study, we preliminarily investigated the biological functions of the mTOR and AMPK signaling pathways in CC. In addition, the therapeutic effects and potential molecular mechanisms of OA in CC cells were also investigated. The results from this study revealed that mTOR was highly expressed in CC patients and acted as an oncogene. The AMPK signaling pathway mediated by mTOR predicted the poor prognosis of CC patients. Furthermore, OA inhibited the proliferation and viability of CC cells. The apoptosis rate of CC cells was clearly increased following OA treatment. In addition, activation of AMPK and inhibition of mTOR were involved in autophagy and apoptosis induced by OA. The anticancer activities of OA are closely related to activation of the AMPK-mTOR signaling pathway, which is crucial in apoptosis and autophagy processes. OA induced apoptosis and autophagy predominantly via an AMPK activation-dependent mechanism in CC cells.

## 2. Materials and Methods

### 2.1. Bioinformatics of mTOR

To explore the interacting genes, proteins, and signaling pathways of mTOR, a pivotal sensor of autophagy and apoptosis, the GeneCards (https://www.genecards.org/) database, was applied [23]. The protein-protein interaction (PPI) network of mTOR was constructed by the Search Tool for the Retrieval of Interacting Genes (STRING, http://string-db.org, version 11.0) [24]. To further investigate the molecular mechanisms of mTOR in CC, the Database for Annotation, Visualization and Integrated Discovery (DAVID, http://david.ncifcrf.gov, version 6.8) was used [25]. Biological information was extracted from the comprehensive set of genes and proteins, which provides functional annotations. Kyoto Encyclopedia of Genes and Genome (KEGG) enrichment analysis was applied to analyze the function and cell signaling pathways of mTOR. Bubble plots of enrichment results were drawn using the “ggplot” package in R software (version 3.6.3, 64-bit, https://www.r-project.org/). *P* < 0.05 was considered statistically significant.

The Gene Expression Omnibus (GEO, http://www.ncbi.nlm.nih.gov/geo) database was applied to obtain prognostic data and matched survival data of CC patients. Least absolute shrinkage and selection operator (LASSO) regression analysis [26] was performed for the prognostic value of target genes by the R package glmnet.

### 2.2. Preparation of Oleanolic Acid and Reagents

Standard of OA (purity ≥ 98%, Cat. no. CHB180311) was purchased from Chroma Biotechnology Co, Ltd. (Chengdu, China). OA was dissolved in dimethyl sulfoxide (DMSO) and diluted to the corresponding concentration when applied to cell lines.

Acadesine (AICAR, Cat. no. HY-13417), a specific agonist of AMPK, and rapamycin (Cat. no. AY-22989), a selective small-molecule inhibitor of mTOR, were obtained from MedChem Express (Shanghai, China). AICAR and rapamycin were dissolved in DMSO and diluted to the indicated concentrations when applied to cell lines.

### 2.3. Cell Lines and Culture

The human-derived COAD cell lines SW-480 (ATA-CL1052) and HCT-116 (CL0125) were purchased from PuJian Cell Center (Wuhan, China) and FengHui Cell Center (Beijing, China), respectively, and the human normal colon epithelial cell line NCM-460 (ATA-CL1041) was purchased from PuJian Cell Center (Wuhan, China). All cell lines were cultivated in Dulbecco's modified Eagle's medium (Gibco, Thermo Fisher Scientific, Inc.) including ten percent fetal bovine serum (Gibco, Thermo Fisher Scientific, Inc.), 1% streptomycin, and penicillin (Thermo Fisher Scientific, Inc.) and then nurtured in 5% CO_2_ at 37°C.

### 2.4. Cell Viability and Proliferation Evaluation

The viability of SW-480 and HCT-116 cells treated with OA for 24 h was assessed by adding 10% (vol/vol) cell counting kit-8 (CCK-8; Lot. PG658, Dojindo, Tokyo, Japan) to the cells and incubating for 15 min at 37°C. Absorbance was measured at 450 nm. Cell viability was calculated as cell viability (%) = 100 × (OD treatment/OD control). For SW-480 and HCT-116 cells, the 50% inhibitory concentration (IC_50_) was calculated.

The proliferation of HCT-116 and SW-480 cells was evaluated by colony formation assay. Five hundred cancer cells per well were seeded into a twelve‐well plate. Then, the cells were cultured with fresh medium containing OA or DMSO. The medium was exchanged every 2 days. Ten days later, colonies were fixed with 4% paraformaldehyde for 10 min, followed by 0.25% crystal violet staining at room temperature.

### 2.5. Fluorescent Staining and Morphological Identification

Cell samples were collected and stained with fluorescent dyes. The main fluorescent dyes included Hoechst 33342 (H3570, Invitrogen) for quantitative cell counts, calcein AM (C3099, Invitrogen) for cell survival tracking, ethidium homodimer-1 (EthD-1) (L3224, Invitrogen) for apoptotic cell tracking, Deep Red Actin Tracking Stain (A57245, Invitrogen) for filamentous actin (F-actin, a cell structure of membranes) marking, and tetramethylrhodamine (TMRM, T668, Invitrogen) as a mitochondrial membrane potential indicator. Morphological identification and quantitative statistics of HCT-116 and SW-480 cells were examined by a high-content system (HCS) array scan (Thermo Scientific, Massachusetts, USA). The parameters and forma settings were reported previously by O'Brien et al. [27] and Yang et al. [23], and the wavelength in different channels was set to collect fluorescent images. The mean fluorescence intensity of cells was analyzed by the Array Scan XTI system through a software algorithm.

### 2.6. Flow Cytometry (FCM) for Apoptosis Analysis

The apoptosis of HCT-116 and SW-480 cells was analyzed by FCM. The Apoptosis Detection Kit (Cat. no. 559763) was purchased from BD (San Jose, CA). Cells for the experiment were collected and washed with cold PBS and then stained with Annexin V/PE and 7-amino-actinomycin (7-AAD) in 400 *μ*l binding buffer. The cells were incubated for 20 min at room temperature, and the apoptosis rate was analyzed by FCM (BD, FACSCanto II, USA).

### 2.7. Real-Time Quantitative PCR for mRNA Expression

Microarray analysis was performed using RNA extracts from SW-480, HCT-116, and NCM-460 cells to validate the expression level of mRNA. TRIzol reagent (Nordic Bioscience, Beijing, China) was applied, and mRNA was converted into cDNA using a reverse transcription kit (Thermo Scientific, USA) according to the manufacturer's protocol. The primer sequences for mTOR, Bcl-2, BAX, caspase-3, caspase-8, and caspase-9 are listed in Table 1. Quantitative real-time PCR for these mRNAs was performed and analyzed using cDNA and SYBR Green PCR Master Mix (Nordic Bioscience, Beijing, China). RT-PCR was performed on a 7500 Fast Real-Time PCR system (Applied Biosystems, Foster City, CA, USA). The relative amounts of mRNA were determined based on 2^−ΔΔCt^ calculations with *β*-actin as an endogenous reference.

### 2.8. Western Blotting (WB)

The WB procedures were performed as previously described by Yang et al. [28]. Details on the main antibodies are as follows: rabbit anti-AMPK-*α* Ab (Proteintech, Cat. no. 66536-1-Ig, dilution: 1 : 1000), rabbit anti-mTOR Ab (Proteintech, Cat. no. 66888-1-Ig, dilution: 1 : 5000), phospho-mTOR (Ser 2448) Ab (Cell Signaling Technology, Cat. no. 2971, dilution: 1 : 500), rabbit anti-TSC2 Ab (Proteintech, Cat. no. 24601-1-AP, dilution: 1 : 500), phospho-TSC2 (Ser 1387) Ab (Cell Signaling Technology, Cat. no. 5584, dilution: 1 : 500), rabbit anti-BAX Ab (Proteintech, Cat. no. 50599-2-Ig, dilution: 1 : 1000), rabbit anti-Beclin 1 Ab (Proteintech, Cat. no. 11306-1-AP, dilution: 1 : 1000), rabbit anti-LC3B-II Ab (Proteintech, Cat. no. 18725-1-AP, dilution: 1 : 500), rabbit anti-ULK1 Ab (Proteintech, Cat. no. 20986-1-AP, dilution: 1 : 1000), Phospho-ULK1 (Ser 317) Ab (Cell Signaling Technology, Cat. no. 37762, dilution: 1 : 1000), and GAPDH monoclonal antibody (Proteintech, 60004-1-Ig, dilution: 1 : 10000). The gray values of the blots in the scanned images were measured using ImageJ Plus software (National Institutes of Health, Bethesda, MD, USA), and GAPDH was set as a loading control for the gray value of each target protein.

### 2.9. Statistical Analysis

All data are presented as the mean ± standard deviation (SD) and were analyzed with the SPSS software program (version 18.0; SPSS Inc., Chicago, IL, USA). Data are presented using *one-way ANOVA* followed by LSD. *P* < 0.05 was considered statistically significant, and *P* < 0.01 was considered highly significant. GraphPad Prism software for Windows (version 8.0; San Diego, CA, USA) was utilized for the visible presentation of all the results.

## 3. Results

### 3.1. mTOR and Its Interacting Genes Were Significantly Enriched in the AMPK Signaling Pathway

**Figure 1:**
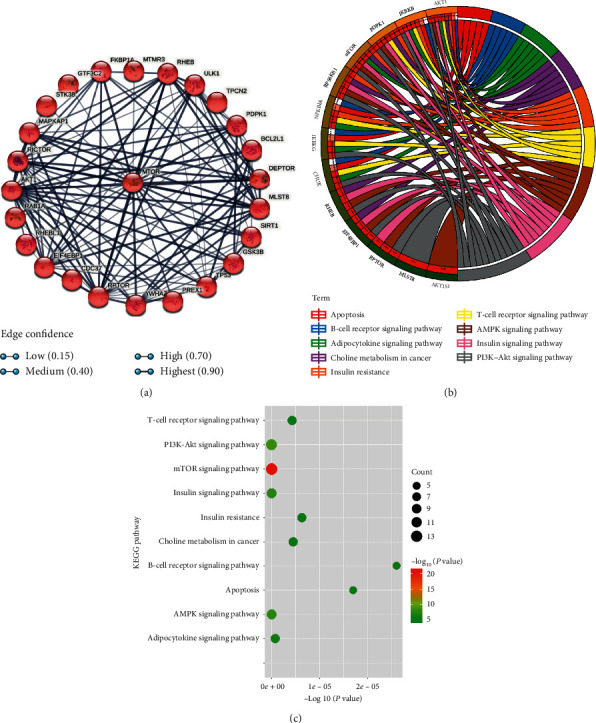
Bioinformatics of mTOR. (a) PPI network of mTOR. (b) The top 10 enriched KEGG pathways and enriched genes presented by circos plot. (c) The top 10 enriched KEGG pathways presented by bubble plot.

There were 2454 genes that interact with mTOR in the GeneCards database. Then, the top 25 interacting genes (AKT1, MLST8, RHEB, RICTOR, RPTOR, BCL2L1, CDC37, DEPTOR, EIF4EBP1, FKBP1A, GSK3B, GTF3C2, MAPKAP1, MTMR3, MTOR, PDPK1, PREX1, RAB1A, RHEBL1, SIRT1, STK38, TP53, TPCN2, ULK1, and YWHAZ) were selected to explore the interacting proteins of mTOR. The STRING database was used to establish a PPI network of mTOR (Figure 1(a)). In total, the enrichment *P* value of the PPI network was <1.0*e* − 16, indicating that the interacting proteins were at least partially connected as a group biologically. KEGG pathway enrichment analysis in the DAVID database was used to explore the interacting genes of mTOR in signaling pathways. The top 10 enriched pathways are presented in Figures 1(b) and 1(c). The interacting genes of mTOR were significantly enriched in the AMPK signaling pathway with an enrichment *P* value = 1.30*e* − 08 (Figure 1(c)). In the AMPK signaling pathway, the enriched genes were mTOR, RPTOR, RPS6KB1, PDPK1, RHEB, AKT1S1, EIF4EBP1, and AKT1 (Figure 1(b)).

### 3.2. AMPK Signaling Pathway Mediated by mTOR Predicted the Poor Prognosis of CC Patients

**Figure 2:**
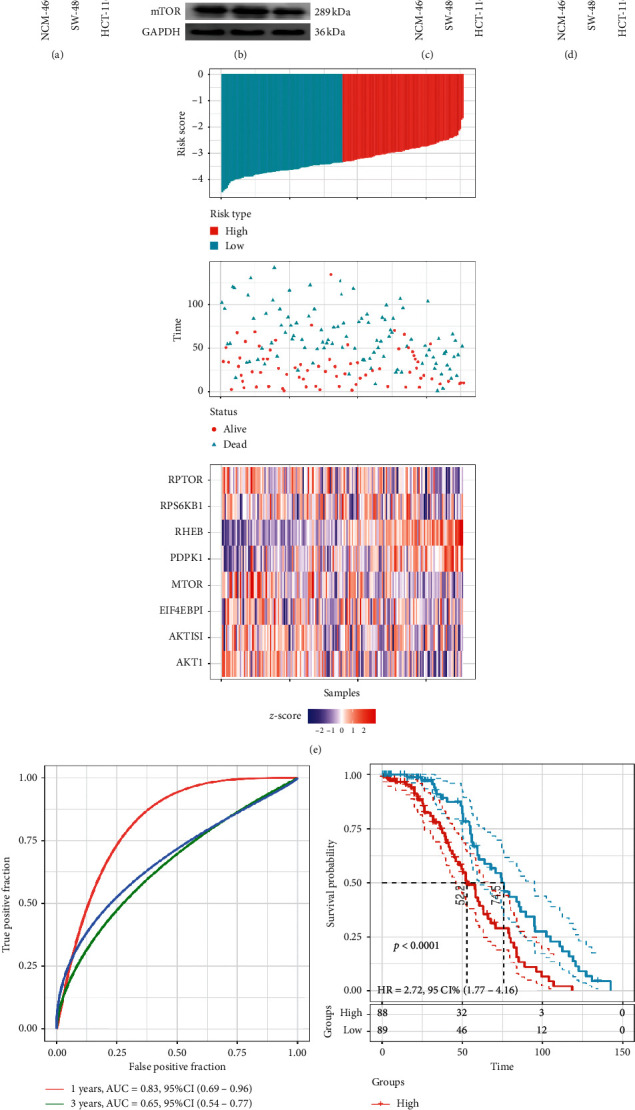
(a) mRNA expressions of mTOR verified by RT-PCR, ^*∗∗*^*P* < 0.01 versus NCM-460 group. (b) Western blotting images of mTOR and p-mTOR (Ser 2448). (c) Relative protein level of mTOR. (d) Relative protein level of p-mTOR; NS: nonsignificant versus NCM-460 group. (e) Risk score, survival status, and the expression of 4 mTOR, RPTOR, RPS6KB1, PDPK1, RHEB, AKT1S1, EIF4EBP1, and AKT1. (f) 1-, 3-, and 5-year ROC analysis of prognosis classification for risk score. (g) KM survival analysis of patients with high-risk score vs. low-risk score. The red curve represents high expression and the blue curve represents low expression.

To validate the expression level of mTOR in the AMPK signaling pathway, the mRNA and protein expression of mTOR was determined by RT-PCR and WB. The results are presented in Figures 2(a)–2(d). The mRNA expression of mTOR in HCT-116 and SW-480 cell lines was significantly higher than that in NCM-460 cells (Figure 2(a)). However, neither the total protein (Figures 2(b) and 2(c)) nor the phosphorylated mTOR (p-mTOR, Figures 2(b) and 2(d)) was nonsignificant versus NCM-460 cells in HCT-116 and SW-480 cell lines. We speculated that the high mRNA expression of mTOR was beneficial to the escape and survival of CC cells, which contributed to the poor prognosis of CC patients [29].

Then, a prognostic risk score was constructed and combined with the clinical information of CC patients by using seven mTOR regulator-associated signatures in the AMPK signaling pathway through LASSO regression analysis. The risk score formula used in the GSE 17536 cohort was as follows: risk score_8_ = −0.9321 ∗ exp^MTOR^ − 0.48157 ∗ exp^RPTOR^ + 0.147918 ∗ exp^RPS6KB1^ + 0.693419 ∗ exp^PDPK1^ + 1.173201 ∗ exp^RHEB^ − 0.08506 ∗ exp^AKT1S1^ − 0.00923 ∗ exp^EIF4EBP1^ − 0.63697 ∗ exp^AKT1^. The results revealed that patients with a high-risk score had significantly lower OS than those with a low-risk score (Figure 2(e)). The increased expression levels of RPS6KB1, PDPK1, and RHEB were associated with high risk, highlighting them as risk factors. Elevated expression of MTOR, RPTOR, AKT1S1, EIF4EBP1, and AKT1 was correlated with low risk, suggesting that they are protective factors. ROC analysis of risk score with the AUCs for predicting 1-, 3-, and 5-year OS were 0.67, 0.65, and 0.83, respectively (Figure 2(f)), indicating that this prognostic model has a high area under the AUC. Finally, *z*-score analysis of risk score was used to categorize samples into the high-risk group (with scores > 0) and the low-risk group (with scores < 0). Eighty-eight samples were classified into the high-risk group, and 89 samples were classified into the low-risk group (Figure 2(g)). KM analysis revealed significant survival differences in the two groups (log rank *P* < 0.0001, HR = 2.72, and 95% CI : 1.77–4.16, Figure 2(g)), indicating that patients in the high-risk group might have a poor prognosis with a shorter OS.

### 3.3. OA Suppressed the Viability and Proliferation of CC Cells in a Dose-Dependent Manner

**Figure 3:**
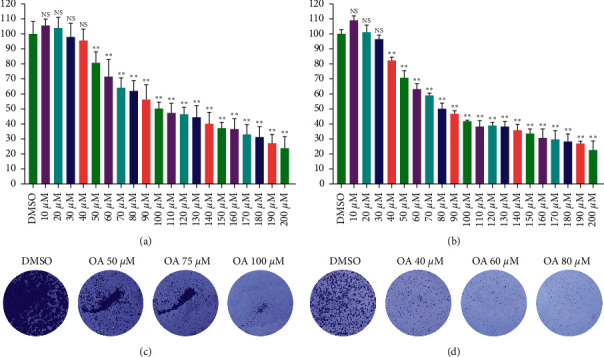
Effects of OA on cell viability and proliferation of HCT-116 and SW-480 cells. (a) HCT-116 cells in different concentrations of OA (10 *μ*Μ–200 *μ*Μ) the activity of cells. (b) SW-480 cells in different concentrations of OA (10 *μ*Μ–200 *μ*Μ) the activity of cells. (c) Colony formation assay of HCT-116 cells. (d) Colony formation assay of SW-480 cells.

The IC_50_ concentrations of OA for HCT-116 and SW-480 cells were explored first. The concentrations of OA were set as 10 *μ*M to 200 *μ*M. As presented in Figure 3(a), 100 *μ*M OA notably inhibited the viability of HCT-116 cells (cell viability was 50.37 ± 4.62), and 80 *μ*M OA remarkably restrained the viability of SW-480 cells (cell viability was 50.10 ± 3.73, Figure 3(b)). In addition, as the concentration of OA increased, the viability of cells gradually decreased. The cell viability of HCT-116 cells treated with 200 *μ*M OA was lower than 75% (23.82 ± 7.73, Figure 3(a)) as well as that of SW-480 cells (22.84 ± 5.92, Figure 3(b)). Accordingly, 100 *μ*M and 80 *μ*M OA were set as the IC50 values for HCT-116 and SW-480 cells, respectively.

The results of the colony formation assay are shown in Figures 3(c) and 3(d). As the concentration of OA increased (50 *μ*M, 75 *μ*M, and 100 *μ*M), the proliferation of HCT-116 cells was significantly reduced (Figure 3(c)). The same results were observed in SW-480 cells. The proliferation ability of SW-480 cells gradually declined with the increasing concentration of OA (40 *μ*M, 60 *μ*M, and 80 *μ*M, Figure 3(d)). These results demonstrated that OA suppressed the viability and proliferation of HCT-116 and SW-480 cells in a dose-dependent manner.

### 3.4. OA Induced Apoptosis in CC Cells via AMPK Activation and mTOR Suppression

**Figure 4:**
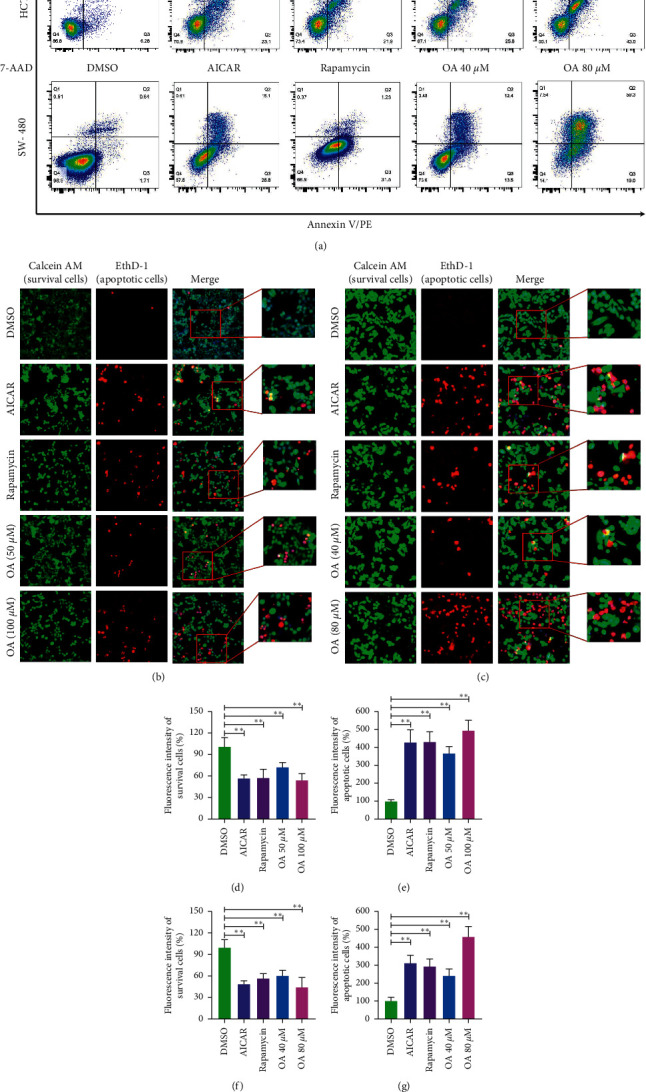
Apoptosis of CC cells induced by AICAR, rapamycin, and OA. (a) Apoptosis rate of HCT-116 and SW-480 cells detected by FCM. (b) Morphological features of HCT-116 cells. Survival cells (green fluorescence) and apoptotic cells (red fluorescence) were reflected by fluorescence staining intensity. (c) Morphological features of SW-480 cells. Survival cells (green fluorescence) and apoptotic cells (red fluorescence) were reflected by fluorescence staining intensity. (d) Survival cells count of HCT-116 cells. (e) Apoptotic cells count of HCT-116 cells. (f) Survival cells count of SW-480 cells. (g) Apoptotic cells count of SW-480 cells. ^*∗∗*^*P* < 0.01 versus DMSO group.

FCM was applied to analyze the apoptosis rate of HCT-116 and SW-480 cells following AICAR, rapamycin, and OA intervention. The results are presented in Figure 4(a). The results indicated that Annexin V/PE-positive cells (both early and late apoptosis) were significantly increased after AICAR (1 mM), rapamycin (1 *μ*M), and OA treatment. AICAR, rapamycin, and OA mainly induced HCT-116 and SW-480 cell apoptosis at the early stage (Figure 4(a)). Thus, we speculated that the activation of AMPK and suppression of mTOR could induce apoptosis of CC cells.

Then, morphological features and quantitative statistics of HCT-116 and SW-480 cells were identified by HCS array. As displayed in Figures 4(b) and 4(c), HCT-116 and SW-480 cells in the DMSO group presented homogeneous calcein, AM, and EthD-1 fluorescence. In contrast, EthD-1 staining showed a significant increase in red fluorescent cells in the AICAR (1 mM), rapamycin (1 *μ*M), and OA groups (50 *μ*M and 100 *μ*M for HCT-116 cells, 40 *μ*M and 80 *μ*M for SW-480 cells). Regarding quantitative statistics, the surviving cell count of HCT-116 cells was significantly decreased (Figure 4(d)), and apoptotic cells were clearly increased (Figure 4(e)) after treatment with AICAR, rapamycin, and OA for 24 h. The same results were observed with SW-480 cells. As presented in Figures 4(f) and 4(g), a decline in surviving cells (Figure 4(f)) and a surge in apoptotic cell counts (Figure 4(g)) were detected. These results suggested that OA may induce apoptosis in CC cells via AMPK activation and mTOR suppression.

### 3.5. OA Stimulated Autophagy in CC Cells by Activating the AMPK-mTOR Signaling Pathway

**Figure 5:**
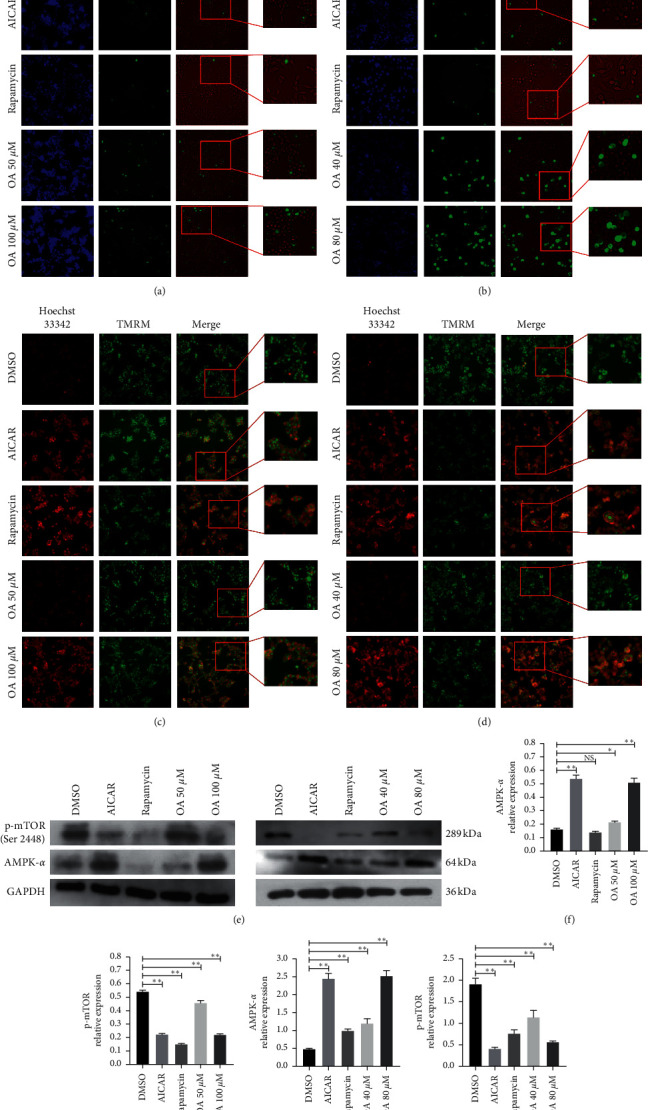
Influences of OA on the F-actin, mitochondrial membrane potential, and protein expression of HCT-116 and SW-480 cells. (a) F-actin identification of HCT-116 cells. Positive expressions were stained by green fluorescence. (b) F-actin identification of SW-480 cells. Positive expressions were stained by green fluorescence. (c) Mitochondrial membrane potential of HCT-116 cells. Nondepolarized membrane potential was stained by green fluorescence; depolarized membrane potential was stained by red fluorescence. (d) Mitochondrial membrane potential of SW-480 cells. Nondepolarized membrane potential was stained by green fluorescence; depolarized membrane potential was stained by red fluorescence. (e) WB images of AMPK-*α* and p-mTOR (Ser 2448). (f) Relative AMPK-*α* protein level in HCT-116 cells. (g) Relative p-mTOR (Ser 2448) protein level in HCT-116 cells. (h) Relative AMPK-*α* protein level in SW-480 cells. (i) Relative p-mTOR (Ser 2448) protein level in SW-480 cells.

In the initial period of cell autophagy, the cell membrane, mitochondria, cytoplasm, and nucleus undergo varying degrees of changes, and these changes can be judged as evidence for evaluating cell autophagy [30, 31]. F-actin, the structure of cell membranes, and mitochondrial membrane potential were assessed in this study. As shown in Figure 5(a), AICAR (the specific agonist of AMPK) and rapamycin (the target inhibitor of mTOR) did not affect F-actin in HCT-116 cells or SW-480 cells (Figure 5(b)), indicating that the target site of AICAR and rapamycin was not the cell membrane. Notably, the injured F-actin was obviously increased in the OA group, especially at concentrations of 100 *μ*M for HCT-116 cells (Figure 5(a)) and 80 *μ*M for SW-480 cells (Figure 5(b)).

As presented in Figure 5(c), the mitochondrial membrane potential was depolarized and decreased in HCT-116 cells following AICAR, rapamycin, and OA intervention. Furthermore, the fluorescence intensity of the cell nucleus marked by Hoechst 33342 was remarkably increased. The same changes were observed in SW-480 cells (Figure 5(d)). These changes indicated that autophagy in HCT-116 cells and SW-480 cells induced by OA, AICAR, and rapamycin mainly occurred in mitochondria.

Then, the expression levels of AMPK-*α* and p-mTOR were examined by WB. Remarkably, the expression of AMPK-*α* proteins was significantly stimulated by AICAR at 1 mM and OA directly in HCT-116 cells and SW-480 cells (Figures 5(e), 5(f), and 5(h), resp.). Meanwhile, the expression level of p-mTOR was notably suppressed by AICAR, rapamycin, and OA in both CC cell lines (Figures 5(e), 5(g), and 5(i), resp.). However, rapamycin at 1 *μ*M did not reduce the expression level of AMPK-*α*. These results revealed that OA could activate AMPK and suppress mTOR (via reducing phosphorylation level), which led to the biological effects of autophagy and apoptosis in CC cells.

### 3.6. OA Regulated the Expression of mRNA Related to Autophagy and Apoptosis in CC Cells

**Figure 6:**
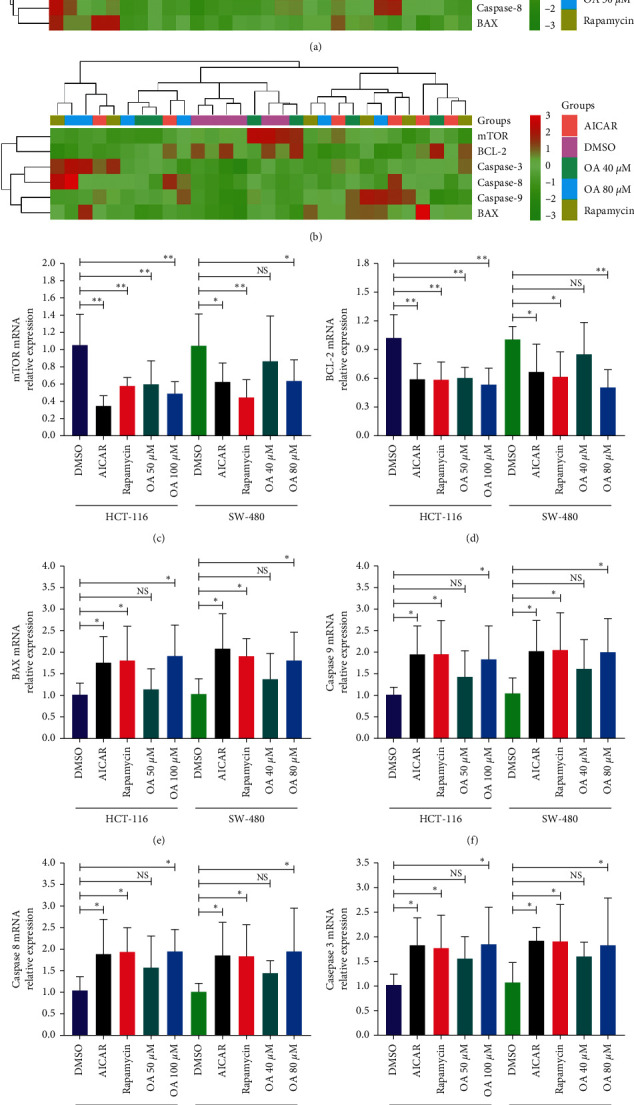
Influences of OA on the mRNA expression of HCT-116 and SW-480 cells. NS: nonsignificant versus DMSO group. ^*∗*^*P* < 0.05 versus DMSO group; ^*∗∗*^*P* < 0.01 versus DMSO group. (a) Heatmap of mRNA expression for HCT-116 cells. (b) Heatmap of mRNA expression for SW-480 cells. (c) mRNA expression of mTOR. (d) mRNA expression of BCL-2. (e) mRNA expression of BAX. (f) mRNA expression of caspase-9. (g) mRNA expression of caspase-8. (h) mRNA expression of caspase-3.

To further clarify the relationship between OA and the AMPK-mTOR signaling pathway in the process of autophagy and apoptosis, the mRNA levels related to autophagy and apoptosis were verified by RT-PCR experiments. The results are presented in Figures 6(a) and 6(b) with a heatmap. The mRNA expression of mTOR (Figure 6(c)), serving as a pivotal sensor of autophagy, and the antiapoptosis gene BCL-2 (Figure 6(d)) were downregulated by AICAR, rapamycin, and OA in HCT-116 and SW-480 cells. Furthermore, the regulator genes of autophagy and apoptosis, including BAX (Figure 6(e)), caspase-9 (Figure 6(f)), caspase-8 (Figure 6(g)), and caspase-3 (Figure 6(h)), were noticeably increased following AMPK and OA intervention. Thus, we speculated that the autophagy and apoptosis induced by OA may be AMPK activation dependent.

### 3.7. OA Triggered the AMPK-mTOR Signaling Pathway in CC Cells in an AMPK Activation‐Dependent Manner

**Figure 7:**
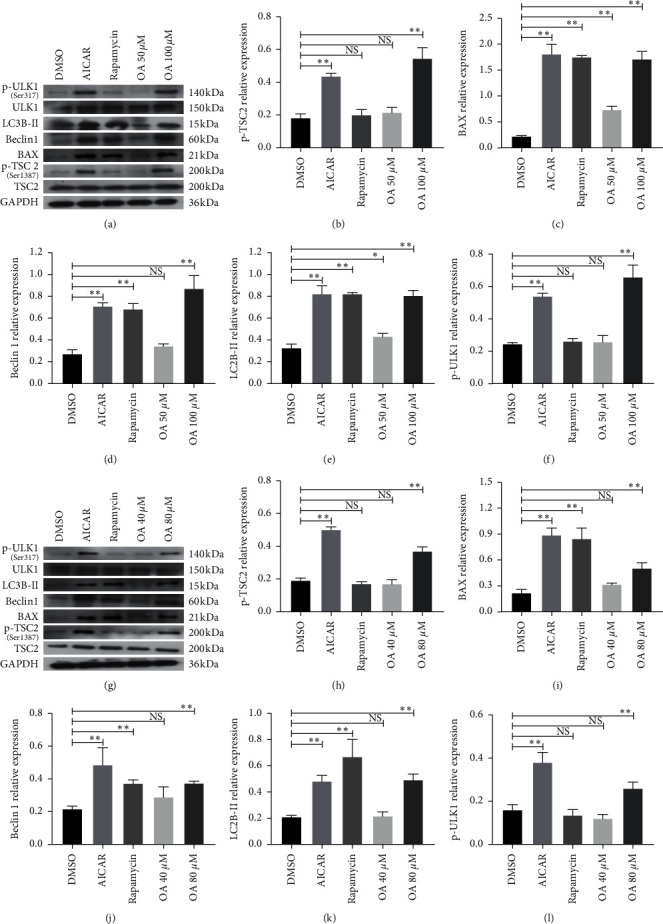
(a) Western blotting images of TSC2, p-TSC2 (Ser 1387), BAX, Beclin 1, LC3B-II, ULK1, and p-ULK1 (Ser 317) in HCT-116 cells. (b) Relative p-TSC2 (Ser 1387) in HCT-116 cells. (c) Relative BAX protein level in HCT-116 cells. (d) Relative Beclin 1 protein level in HCT-116 cells. (e) Relative LC3B-II protein level in HCT-116 cells. (f) Relative p-ULK1 (Ser 317) protein level in HCT-116 cells. (g) Western blotting images of TSC2, p-TSC2 (Ser 1387), BAX, Beclin 1, LC3B-II, ULK1, and p-ULK1 (Ser 317) in SW-480 cells. (h) Relative p-TSC2 protein level in SW-480 cells. (i) Relative BAX protein level in SW-480 cells. (J) Relative Beclin 1 protein level in SW-480 cells. (k) Relative LC3B-IIprotein level in SW-480 cells. (l) Relative p-ULK1 (Ser 317) protein level in SW-480 cells. NS: nonsignificant versus HCT-116 and SW-480 group; ^*∗*^*P* < 0.05 versus HCT-116 and SW-480 group; ^*∗∗*^*P* < 0.01 versus HCT-116 and SW-480 group.

The downstream proteins in the AMPK-mTOR signaling pathway were estimated by WB experiments. The protein expression of HCT-116 cells and SW-480 cells is presented in Figures 7(a) and 7(g), respectively. The total protein expression of TSC2, a direct target of AMPK, was not significantly different between the groups. However, the phosphorylation level of TSC2 (p-TSC2) was increased by AICAR and OA in HCT-116 cells (Figure 7(b)) and SW-480 cells (Figure 7(h)). Of note, p-TSC2 remained at a lower level in the rapamycin group. Furthermore, followed by activation of AMPK and inhibition of mTOR and OA intervention, the expression of autophagy-related proteins, including BAX (Figures 7(c) and 7(i)), Beclin 1 (Figures 7(d) and 7(j)), and LC3B-II (Figures 7(e) and 7(k)), was clearly increased in HCT-116 cells and SW-480 cells. In addition, the phosphorylation level of ULK1 (p-ULK1), a regulatory protein of autophagy, was upregulated by AICAR and OA (Figures 7(f) and 7(l)) in HCT-116 cells and SW-480 cells, respectively. Compared with AICAR and OA at high concentrations (100 *μ*M for HCT-116, 80 *μ*M for SW-480), p-ULK1 remained at a lower level in rapamycin and OA at low concentrations (50 *μ*M for HCT-116, 40 *μ*M for SW-480). These results demonstrated that OA might trigger the AMPK-mTOR signaling pathway and induce autophagy in CC cells in an AMPK activation‐dependent manner.

## 4. Discussion

This study illustrated the biological functions of the mTOR and AMPK signaling pathways in CC processes. The results revealed that mTOR was highly expressed in CC patients and acted as an oncogene, which contributed to the poor prognosis of CC patients. In addition, the anticancer activities of OA in human CC cells were identified in this study. Our results confirmed that OA could promote autophagy and apoptosis in CC cells. Further exploration indicated that activation of AMPK and inhibition of mTOR were involved in autophagy and apoptosis induced by OA. The anticancer activities of OA are closely related to activation of the AMPK-mTOR signaling pathway, which is crucial in apoptosis and autophagy processes. OA induced autophagy and apoptosis predominantly in CC cells in an AMPK activation-dependent manner.

Autophagy is an intracellular catabolism system that transports proteins and organelles to lysosomes for degradation and maintains cell homeostasis under stress. According to the degradation pathway, autophagy can be divided into three types: microautophagy, giant autophagy, and partner-mediated autophagy. In general, autophagy is defined as macroscopic autophagy [32]. The process of autophagy is roughly divided into four steps: autophagosome initiation, nucleation, autophagy membrane formation, and degradation of macromolecular products [33]. In the initial step, rapamycin complex 1 (mTORC1) and adenosine monophosphate- (AMP-) activated protein kinase (AMPK) cooperatively activate the autophagy-related gene UNC-51-like kinase 1 (ULK1). During the nucleation of autophagosomes, the ULK1 complex activates and phosphorylates the Beclin 1/Vps34 complex. Then, activation and nucleation-related proteins are involved in the formation of autophagy vesicles [34, 35]. During the formation of autophagosomes, the fatty form of LC3 is transformed into a soluble form (LC3-I) with the degradation of macromolecular contents. Autophagy is regulated by a variety of pathways and molecules. It is well known that the AMPK/mTOR transduction pathway negatively regulates autophagy. mTORC1 responds to changes in the cellular environment and nutritional levels through its upstream negative regulatory molecule (tumor suppressor TSC1/TSC2) [36]. In addition, AMPK, a cellular energy receptor, is activated at low ATP levels. TSC2 is phosphorylated by activated AMPK, which upregulates the GAP activity of TSC1/2 [37]. In mammalian cells, mTORC1 phosphorylates ULK1 and blocks the interaction between AMPK and ULK1, thereby inhibiting autophagy [38]. When the energy supply is insufficient, AMPK can directly phosphorylate ULK1 to promote autophagy. As a survival mechanism, the role of autophagy in maintaining cell homeostasis is self-evident, and its dysfunction is related to many diseases. Generally, autophagy plays a dual role in tumors. On the one hand, autophagy can inhibit growth and invasion; on the other hand, it helps tumor cells survive and escape under stress, especially in the case of apoptosis deficiency. Therefore, autophagy and apoptosis usually exist at the same time, and autophagy is accompanied by apoptosis [39]. In CC, the crosstalk relationships between autophagy and apoptosis involve a variety of signal transduction pathways and regulatory factors, and the AMPK/mTOR signaling transduction pathway is an important way to regulate autophagy and apoptosis. Whether autophagy induces or inhibits apoptosis depends on the cell type, nature and duration of stimulation, or stress [40]. During autophagy initiation, mTORC1 and AMPK cooperatively activate ULK1 to cope with changes in the cellular environment and nutritional levels, and ULK1 is the central component of autophagy. Therefore, the relative activity of AMPK/mTOR in cancer cells plays a critical role in the initiation of autophagy and apoptosis.

Enhanced effects of autophagy could lead to apoptosis of cancer cells, which is beneficial to the treatment of cancers. In this study, we demonstrated that OA, a natural pentacyclic triterpene carboxylic acid, effectively activated AMPK and inhibited mTOR, leading to autophagy and apoptosis activation in CC cells. The results from this study confirmed that the antiapoptotic regulators and biomarkers, mTOR and Bcl-2, were downregulated by AMPK activation and mTOR inhibition, inducing enhanced apoptosis. Meanwhile, the autophagy and apoptosis regulators in the cell apoptosis process, BAX, caspase-3, caspase-8, and caspase-9, were significantly increased under the intervention of OA. These changes suggested that AMPK activation and mTOR inhibition induced by OA promoted autophagy and apoptosis in a crosstalk-dependent manner. Furthermore, the phosphorylation level of TSC2 (p-TSC2) and ULK1 (p-ULK1), a direct supervisor of autophagy, and the downstream targets of AMPK, including BAX, Beclin 1, and LC3B-II, were upregulated followed by activation of AMPK, instead of inhibition of mTOR. Thus, we speculated that autophagy and apoptosis induced by OA may occur in an AMPK-dependent manner.

Overall, the conclusions of this study indicate that OA effectively activates AMPK and inhibits mTOR. OA induces autophagy and apoptosis in CC cells by initiating the AMPK/mTOR signaling pathway. Therefore, it is conceivable that OA could be developed into a potent agent for use against CC in clinical practice.

## Figures and Tables

**Table 1:** Primers sequences of real-time PCR analyses for mRNA expression.

Genes	Forward	Reverse
mTOR	CTTGCTGAACTGGAGGCTGATGG	CCGTTTTCTTATGGGCTGGCTCTC
Bcl-2	TACGAGTGGGATGCGGGAGATG	CCGGGCTGGGAGGAGAAGATG
BAX	GATGCGTCCACCAAGAAGCTGAG	CACGGCGGCAATCATCCTCTG
Caspase-3	GTGGAGGCCGACTTCTTGTATGC	TGGCACAAAGCGACTGGATGAAC
Caspase-8	CGGATGAGGCTGACTTTCTGCTG	GGCTCTGGCAAAGTGACTGGATG
Caspase-9	GACCAGAGATTCGCAAACCAGAGG	AAGAGCACCGACATCACCAAATCC
*β*-Actin	GGCCAACCGCGAGAAGATGAC	GGATAGCACAGCCTGGATAGCAAC

## Data Availability

The data used to support the findings of this study are included within the article.
